# Pathogenetic determinants in Kawasaki disease: the haematological point of view

**DOI:** 10.1111/jcmm.12992

**Published:** 2017-01-07

**Authors:** Domenico Del Principe, Donatella Pietraforte, Lucrezia Gambardella, Alessandra Marchesi, Isabella Tarissi de Jacobis, Alberto Villani, Walter Malorni, Elisabetta Straface

**Affiliations:** ^1^ Department of Therapeutic Research and Medicine EvaluationSection of Cell Aging and Gender MedicineIstituto Superiore di SanitàRomeItaly; ^2^ Department of NeurosciencesSection of Cell Aging and Gender MedicineIstituto Superiore di SanitàRomeItaly; ^3^ General Pediatric and Infectious Disease UnitInternal Care DepartmentBambino Gesù Children's HospitalRomeItaly

**Keywords:** Kawasaki disease, etiopathogenesis, infection, immunity, biomarkers, genetics, haematological features

## Abstract

Kawasaki disease is a multisystemic vasculitis that can result in coronary artery lesions. It predominantly affects young children and is characterized by prolonged fever, diffuse mucosal inflammation, indurative oedema of the hands and feet, a polymorphous skin rash and non‐suppurative lymphadenopathy. Coronary artery involvement is the most important complication of Kawasaki disease and may cause significant coronary stenosis resulting in ischemic heart disease. The introduction of intravenous immunoglobulin decreases the incidence of coronary artery lesions to less than 5%. The etiopathogenesis of this disease remains unclear. Several lines of evidence suggest that an interplay between a microbial infection and a genetic predisposition could take place in the development of the disease. In this review, we summarize the state of the art of pathogenetic mechanisms of Kawasaki disease underscoring the relevance of haematological features as a novel field of investigation.

## Introduction

In the 1970s, Tomisaku Kawasaki described the clinical features of a new illness called mucocutaneous lymph node syndrome. This disease presented a small percentage (1.7%) of sudden death, which often took place after 2–3 weeks of fever 1. Most of these deaths were the result of thrombotic occlusion of coronary artery, with aneurysmal rupture occurring in ~15% of fatalities. Currently, this disease, referred as to Kawasaki disease (KD), is known to predominantly affect young children (80% of cases occur between 6 months and 4 years) with a male predominance (approximately 1.2–1.7 times commoner) 2, 3.

The incidence of KD varies considerably between ethnic groups. In North East Asia it is 20 times higher than in Caucasia. The highest incidence is reported in Japan 3, 4. A recent study on the KD epidemiology point out that in rapidly industrializing countries, such as China and India, the disease may replace rheumatic heart disease (the most common cause of acquired heart disease in children) 5. Instead, in Western Australia, KD epidemiology is similar to the European‐Caucasian populations 6. The etiopathogenesis of KD has not yet been identified. It can be caused by infectious agents 7, environmental toxins 8, genetic factors 9 or by a superantigen that causes non‐specific activation of T cells 10, 11.

Some progress improving our knowledge on the pathogenesis of this disease has recently been achieved. In this review, we report recent data suggesting a scenario in which dysregulation of platelet–leucocyte interaction and T‐helper type 17/CD4^+^CD25^+^ regulatory T cells (TH17/T_regs_) may function as immunological and inflammatory amplifiers resulting in the defective inflammation resolution in KD patients.

## Kawasaki disease features

As stated above, although 45 years have passed from the first description, the exact etiopathogenesis of KD remains unknown. Some clinical and epidemiologic lines of evidence support the implication of infectious agents 7, 12, 13, 14. Some pathologic studies proposed an acute coronary arteritis followed by healing, but they failed to account for the complex vasculopathy and clinical course of KD. In addition, some advances in molecular genetic analysis and completion of the human genome project led to identify some genes associated with KD. These findings will be discussed below.

### Etiopathogenesis

In the 2011, Rowley and collaborators 13 revealed the presence of a marked IgA plasma cell response in acute KD tissues, suggesting a specific immune response potentially directed at antigens that have been suggested to be involved in disease etiopathogenesis. Indeed, the use of synthetic antibodies derived from oligoclonal IgA gene sequences has identified intracytoplasmic inclusion bodies with virus‐like particles in intact tissue sections from children with KD, specifically in the ciliated epithelium of medium‐sized bronchi 13. These studies suggested, therefore, a putative viral aetiologic agent as a cause of systemic vasculitis in KD patients. Moreover, pathologic studies identified three distinct, but linked basic KD vascular processes: necrotizing arteritis, subacute chronic vasculitis and luminal myofibroblastic proliferation 15. Necrotizing arteritis is an acute synchronized neutrophilic process of medium‐sized arteries that could be responsible for the saccular aneurysms that can lead to the formation of thrombi or ruptures within the first month. It starts at the endothelium of medium‐sized muscular and elastic arteries and progresses peripherally. Luminal myofibroblastic proliferation occurs in close association with subacute chronic vasculitis. It is a unique process that involves medial (smooth muscle cell‐derived) myofibroblasts and their matrix products, which form a concentric mass that can progressively obstruct the arterial lumen. Both subacute chronic vasculitis and luminal myofibroblastic proliferation can persist for months to years following the onset of KD. Furthermore, giant aneurysms are most likely to develop stenosis resulting from repetitive layering of thrombi, which eventually become occlusive, whereas dilated coronary arteries that preserve some portion of the media most likely undergo luminal myofibroblastic proliferation with or without thrombosis 15.

Some further insight could derive from the study of some haematological features encountered in these patients. For instance, the formation of heterotypic platelet–leucocyte aggregates, which is dependent on platelet activation 16, cannot be ruled out. Leucocyte–RBC–platelet aggregates could at least partially be associated with the release of pro‐aggregating factors, *e.g*. arachidonate 17, and/or with changes in cell surface molecules expression, including P‐selectin. This crosstalk between activated platelets and leucocytes operates through several systems, including the interaction of P‐selectin with P‐selectin glycoprotein ligand‐1 (PSGL‐1). P‐selectin and PSGL‐1 are vascular adhesion molecules that play an important role in the inflammatory response by mediating the interaction of leucocytes with stimulated endothelium and platelets bound in the vicinity of vascular injury. P‐selectin captures leucocytes from the blood to bring them into contact with the endothelial cell surface on the blood vessel wall, where P‐selectin–PSGL‐1 interaction supports leucocyte rolling 18. On the other hand, neutrophils can interact with adherent platelets and leucocytes in a process called secondary capture, which is often followed by neutrophil–endothelial interactions. During inflammation neutrophils recruited by platelets contribute, through Toll‐like receptor 4, to neutrophil extracellular traps (NETs) formation that function as a barometer for systemic infection 19. Importantly, while platelets stimulate NET formation, NETs cause platelet activation and aggregation, thus linking inflammation and thrombosis 20. The occurrence of circulating platelet–neutrophil aggregates 21, the alteration of fibrinogen cascade 22 and its content 23, previously observed in patients with KD, seem to support the relevance of this mechanism in the pathogenesis of the disease.

### Genetics

For decades, KD researchers attempted to identify candidate genes conferring susceptibility to the illness. In particular, genes related to innate and acquired immune functions or to vascular remodelling have been studied 24. Although incidence of KD varies in different ethnic population, genetic polymorphisms of 1,4,5‐trisphosphate 3‐kinase C (*ITPKC*) and caspase 3 (*CASP3)* have been shown to associate with coronary artery lesions formation in both Japanese and Taiwanese populations of patients with KD 25. *ITPKC* gene is located on chromosome 19q23 and acts as a negative regulator of T‐cell activation. *CASP3* gene, located on chromosome 4q35, is profoundly related to the apoptosis of immune cells. Both *ITPKC* and *CASP3* are involved in Ca^2+^/nuclear factor of activated T‐cells pathways, indicating the potential role of this signalling pathway in immune system 25. Furthermore, in 2011, Shimizu *et al*. suggested that genetic variations in the transforming growth factor‐β (TGF‐β) signalling pathway influenced the susceptibility to the manifestation of KD, to the disease outcome as well as to the response to therapy 26. In the immune system, TGF‐β modulates the balance of pro‐inflammatory/anti‐inflammatory T cells, through a complex set of interactions. Genetic variations in the TGF‐β pathway may lead to an imbalance of pro‐inflammatory and regulatory T cells (T_reg_) by affecting the expression of the forkhead/winged helix transcription factor P3 (FOXP3) that is involved in the differentiation, function and survival of CD4^+^CD25^+^ regulatory T cells.

In 2013, the International Kawasaki Disease Genetics Consortium performed a replication study in populations of Korean and European descent and confirmed the genetic contribution to KD pathogenesis by finding an association between *B‐lymphoid tyrosine kinase* (*BLK*) gene with KD 27. *BLK* is a Src family tyrosine kinase expressed primarily in the B‐cell lineage. It is located on chromosome 8p22‐23 and its expression pattern during the acute and convalescent stages of KD correlates with the percentage of B cells in the peripheral blood mononuclear cells. Moreover, Hsueh *et al*. reported an association between the interleukin (IL)‐10 promoter polymorphism at position 592 (*IL‐10*‐592*A allele) and KD susceptibility in the Taiwanese patient population 28. *IL‐10,* located on chromosome 1q31‐q32, is a potent cytokine that exerts pleiotropic effects on immunoregulation and inflammation. An association between the *IL‐10* genetic polymorphisms and coronary artery aneurysms in Taiwanese KD patients was found 29.

Several further evidence suggested that genetic determinants could contribute to the pathogenesis or progression of KD, such as: (*i*) the relationship found between interleukin IL‐1 polymorphisms and initial IVIG treatment failure in KD children 30, (*ii*) the finding that *KCNN2* gene, a member of the KCNN family of potassium channel genes located on chromosome 5q22.3, could have a role as a new risk locus for coronary aneurysms 31, (*iii*) the genome‐wide association studies suggesting *FCGR2A* gene, encoding for low‐affinity immunoglobulin gamma Fc region receptor II‐a protein, as a susceptibility locus for KD onset 32 and, finally, (*iv*) the hypothesis that genetic variations at the human leucocyte antigen (HLA) and killer immunoglobulin‐like receptors (KIR) loci could exert a modulatory activity either individually or in combination (HLA‐KIR epistasis) 33. Candidate genes in the etiopathogenesis of KD are listed in Table 1.

**Table 1:** Candidate genes on the etiopathogenesis of Kawasaki disease

Candidate genes	Locus	Population	Action	Clinical findings in KD	References
*ITPKC*	19q23	Japanese Taiwanese	Negative regulator of T‐cell activation	Coronary artery lesions	17
*CASP3*	4q35	Japanese Taiwanese	Apoptosis of immune cells	Coronary artery lesions	17
*TGF‐*β	19q13.1	European	Modulates the balance of pro‐inflammatory/anti‐inflammatory T cells	Coronary artery lesions	18
*BLK*	8p22‐23	Korean European Japanese	Encodes B‐lymphoid tyrosine kinase	Mechanism in KD pathogenesis unknown	29
*IL‐10*	1q31‐q32	Taiwanese	Exerts pleiotropic effects on immunoregulation and inflammation.	Coreoronary artery aneurysms	52
*KCNN2*	5q22.3	Korean	Encode small‐conductance Ca2+‐activated K+ channels	Coronary artery aneurysms	23
*FCGR2A*	1q23	Korean Asiatic	Encoding for low‐affinity immunoglobulin gamma Fc region receptor II‐a protein	Immune response to intravenous immunoglobulin treatment	24
*KIR*	19q13.4	Asiatic Caucasian	Regulates the innate immune response against pathogens and tumour cells	KD susceptibility	25

*ITPKC*: 1,4,5‐trisphosphate 3‐kinase C; *CASP3*: caspase 3; TGF‐β: transforming growth factor‐β; *BLK*: B‐lymphoid tyrosine kinase; *KCNN2*: potassium calcium‐activated channel subfamily N member 2; *FCGR2A*: Fc fragment of IgG receptor IIa; KIR: killer immunoglobulin‐like receptors.

### Immunity

The activation of the immune system and the cascade of inflammatory factors are considered as important features of KD. A large number of T cells, mononuclear cells, macrophages and plasma cells, with a smaller number of neutrophils, are observed in various organ tissues of fatal cases of acute KD 34. An imbalance of Th1 and Th2 subsets during the acute stage of KD has been suggested by Matsubara *et al*. 35. In particular, a decrease in the numbers of Th1, producing interferon (IFN)‐γ, but not of Th2, producing IL‐4, has been observed. As a general rule, Th1 cells play an important role in cellular immunity by secreting IL‐2 and IFN‐γ*,* whereas Th2 cells have the helper function for the development of antibody‐producing B cells by secreting various cytokines such as IL‐4, IL‐5, IL‐6 and IL‐10. Some of these cytokines play an important role in the progression from systemic activation of the immune system to local inflammation in coronary vessels. Recently, it has been demonstrated that KD patients may be non‐responsive to IVIG when, after IVIG treatment, the serum levels of IL‐6 and IL‐10 decrease slowly and the levels of IL‐4 and TNF‐α increase 36.

Very important are also the data obtained from the study of Th17 and T_reg_ cells. These cells have been described as two subsets distinct from Th1 and Th2 cells. While Th1 and Th2 cells have long been known to regulate cellular and humoural immunity, Th17 cells have been identified only recently as a Th lineage that regulates inflammation *via* production of distinct cytokines, such as interleukin IL‐17. Conversely, T_reg_ cells expressing FOXP3 have an anti‐inflammatory role and maintain tolerance to self‐components by contact‐dependent suppression or releasing anti‐inflammatory cytokines, *e.g*. IL‐10 and TGF‐β1 37. An imbalance between Th17 and T_regs_ cells has been described in the peripheral blood from patients with KD 38. Interestingly, Ikeda and collaborators 39 reported that, in the acute phase, peripheral blood mononuclear cells from KD patients show a unique activation state with a high expression of genes for damage‐associated molecular pattern molecules (S100A9 and S100A12) and a low expression of pro‐inflammatory cytokine genes.

Several further studies pointed at the role of growth factors in the pathogenesis of KD. For example, TGF‐β signalling has been suggested to contribute to the induction of T_regs_ and to aneurysm formation by promoting the generation of myofibroblasts. These cells could mediate the damage to the arterial wall through the recruitment of pro‐inflammatory cells 40. Furthermore, also hepatocyte growth factor, together with VEGF, might play an important role. Their serum levels could be a powerful predictor for the development of coronary artery lesions in KD 41. It must, however, be stressed that, although KD is surely characterized by a marked activation of the immune system with elevations of serum pro‐inflammatory cytokines and chemokines, mainly in the acute phase, the major sources for these chemical mediators remain yet controversial. T cells involved in KD pathogenesis and progression are listed in Table 2.

**Table 2:** T cells involved in Kawasaki disease

Cells	Functions	Clinical findings in KD	References
Th1	Regulate cellular immunity by secreting IL‐2 and IFN‐γ	Down‐regulated	27
Th2	Regulate humoural immunity by secreting IL‐4, IL‐5, IL‐6 and IL‐10	Involved in the response to IVIG treatment	28
Th17	Regulate inflammation by secreting IL‐17	Up‐regulated	29
T_reg_	Anti‐inflammatory role Release IL‐10 and TGF‐β1	Down‐regulated	29

T_reg_: regulatory T cells.

### Infection

Although still controversial, a possible involvement of superantigens in the pathogenesis of KD has been suggested. Both staphylococcal and streptococcal superantigens have been proposed as aetiological agents in the pathogenesis of the disease. Superantigens are extracellular protein toxins whose properties include pyrogenicity, mitogenic activity for specific T‐cell subsets and the ability to increase host susceptibility to endotoxic shock and suppress immunoglobulin. Multiple studies have shown the involvement of variable region of the beta 2 chain (Vb2) in T cells from patients with acute KD 10. An increase in anti‐streptococcal pyrogenic exotoxins types C (SpeC) antibodies has been found in the sera of KD patients in acute phase by Yoshioka *et al*. 42. Moreover, an increase in anti‐SpeC and ‐SpeA IgM in the first few weeks following the illness has been found by Matsubara *et al*. 43. However, despite this evidence, some serological studies found no significant differences in superantigen antibody 44, 45.

A number of viral agents have also been found in the blood from patients with KD, and some of these, *e.g*. coronavirus (HCoV) NL63, have been suggested to exert a pathogenetic role (for a review see Reference 14). However, no definitive conclusion has been drawn as concern the specific association of these agents with KD onset or progression. In fact, some important studies 46, 47 demonstrated that the presence of these infectious agents did not affect the efficacy of IVIG treatment as well as the cardiovascular outcome. The presence of these infectious agents could instead ignite an imbalance of the immune system and lead to the hyperimmune reaction occurring in patients with KD.

### Diagnostic and prognostic biomarkers in KD

Studies carried out on the peripheral blood from patients with KD provided a huge series of data of relevance in the clinical practice as they identified a number of possible biomarkers of disease onset or progression. Among these, biomarkers of inflammation, such as erythrocyte sedimentation rate, C‐reactive protein, haptoglobin, white blood cell counts, fibrinogen‐related proteins, α‐1‐antitrypsin, clusterin, N‐terminal pro‐hormone of brain natriuretic peptide (NT‐proBNP), cardiac troponin I and creatinine kinase have been identified 22, 48. The importance of NT‐proBNP has recently been reassessed, as the levels of this pro‐hormone are raised in both symptomatic and asymptomatic patients with LV dysfunction 23, allowing the specificity of this biomarker for KD patients to be investigated in more detail. Finally, genetic polymorphisms have also been considered as potential biomarkers for KD, with particular regards to six HLA class I genes, two MHC class I chain‐related gene A (MICA) alleles and ITPKC gene 48.

Oxidative stress, linked to inflammation characterizing the disease, has recently been included among the potentially useful diagnostic biomarkers in the vasculature of KD. In particular, changes in the serum redox state 49, increased expression of inducible nitric oxide synthase (NOS) in monocytes/neutrophils 48, as well as elevated concentrations of plasma nitric oxide 50 have been found in KD patients. In fact, oxidative/nitrative stress has been measured in KD inflamed blood vessels, as revealed by the high levels of reactive oxidizing species (*i.e*. superoxide anion and nitric oxide), protein 3‐nitrotyrosine and MPO levels in the blood 51. Protein 3‐nitrotyrosine is the covalent post‐translational modification identifying the formation of nitric oxide‐derived nitrating agents used as a specific biomarker of oxidant burden in many pathological conditions from acute to chronic diseases. Increased levels of MPO, the pro‐inflammatory enzyme released from activated leucocytes, could support the pro‐inflammatory status forming highly oxidizing species and protein oxidation products, such as 3‐nitrotyrosine, promoting lipid peroxidation and inducing an autoimmune response mediated by MPO autoantibodies 52. Moreover, MPO could promote a pro‐coagulant state of the blood, although it is binding at the platelet and neutrophil surface, then favouring the appearance of platelet–neutrophil aggregates detected in KD 21. In this regard, 3‐nitrotyrosine and MPO could play a pathogenetic role in the cardiovascular complications of KD and could be considered as biomarkers of inflammation in this disease.

Furthermore, it has been suggested that this pro‐oxidant blood status could alter also red blood cell (RBC) homeostasis, resulting in a sort of premature ageing in these circulating cells that could lead to anaemia and formation of blood clots 51. In particular, decreased glycophorin A and CD47 expression as well as the externalization of phosphatidylserine (PS) were measured in RBCs from KD patients 51. Glycophorin A, a glycoprotein widely expressed at the RBC surface, is down‐regulated during cell senescence; CD47, an integrin‐associated protein known as the thrombospondin receptor, acts as a marker of self; PS, a phospholipid normally localized to the inner leaflet of the plasma membrane, is externalized to the outer leaflet during cell remodelling, leading to RBC ageing and cell death. The appearance of these ageing and death biomarkers on RBCs from KD patients was related with clinical evaluations. Indeed, during the first 5 days of hospitalization, the number of RBCs and the values of haemoglobin, the mean corpuscular volume and the haematocrit were significantly decreased in KD patients 51. This could be of relevance as RBCs can act as physiological scavengers of ROS in circulating blood and can be considered as ‘biosensors’ for monitoring oxidative imbalance in inflammatory diseases 53. Importantly, alterations in RBC structure and function may independently and synergistically impair blood flow and induce vascular occlusion, whereas premature ageing of RBCs, and their consequent removal from circulation, might be a risk factor for anaemia. Both vascular occlusion and anaemia are pathological conditions found in KD patients 54.

Patients with KD also experience a number of further haematological abnormalities. In the acute phase of the disease, thrombocytosis was often observed. This can exert a pathogenetic role in the cardiovascular complications that characterize KD. Recently, it has been suggested that thrombocytosis in KD could depend on platelet stimulation and defective apoptosis, probably bolstered by nitrosative stress 55. This hypothesis is supported by the detection of circulating platelets–neutrophils aggregates in KD patients 21 and by the increase in markers of platelet activation, such as the shedding of P‐selectin and the externalization of PS. P‐selectin is a cell‐adhesion molecule constitutively expressed in the α‐granules of resting platelets that translocates at the surface during platelet activation. Its release as a shedding phenomenon modulates leucocyte adhesion to both platelets and endothelial cells during inflammatory responses and thrombus formation 56. The externalization of PS in platelets, *i.e*. its presence on outer leaflet of the plasma membrane, is usually associated with a sort of programmed cell death 57, but it has also been correlated with their hyperactivation 58. Importantly, specific studies on platelet activation identified two different platelet subpopulations in the peripheral blood of naïve patients with KD: (*i*) platelets with PS externalization (annexin V positive platelets), characterized by the loss of mitochondrial membrane potential (considered as pro‐coagulant), and (*ii*) platelets without PS externalization (annexin V negative platelets), characterized by mitochondrial membrane hyperpolarization. This latter subpopulation become pro‐coagulant when stimulated by mediators normally released from activated platelets, *e.g*. adenosine diphosphate 59. These two subpopulations may considerably affect the coagulation cascade and inflammatory response in KD patients.

Some further significant alterations have also been detected in plasma from KD patients: (*i*) decreased antioxidant power; (*ii*) decreased levels of asymmetric dymethylarginine (ADMA) and (*iii*) increased levels of soluble P‐selectin and soluble annexin V 55. Increased levels of ADMA, a specific endogenous inhibitor of the endothelial NOS, induce dysfunction of the endothelium, which becomes clinically evident by impaired endothelium‐dependent vasodilation, hyperaggregability of platelets and enhanced monocyte adhesion. Soluble P‐selectin, a cell‐adhesion molecule considered as a marker of *in vivo* platelet activation, has been associated with thrombus growth. Soluble annexin V, released by endothelial cells or platelets, is a calcium‐dependent protein. Its activity is based on phosphatidylserine binding and includes membrane damage repair 60. Peripheral blood diagnostic and prognostic biomarkers are listed in Table 3.

**Table 3:** Diagnostic and prognostic biomarkers in peripheral blood from KD

	Specificity	Clinical findings in KD
Plasmatic biomarkers		
NT‐proBNP		Increased levels in both symptomatic and asymptomatic patients with left‐ventricular dysfunction
MPO	Inflammatory biomarker	Increased concentrations
3‐Nitrotyrosine	Inflammatory biomarker	Increased concentrations
ESR	Inflammatory biomarker	Increased values
PAP	Marker of the antioxidant power plasma	Decreased
ADMA	Endogenous inhibitor of the endothelial NOS	Decreased
sP‐selectin	Marker of platelet activation. It is associated with thrombus growth	Increased
sAnnexin V	Anticoagulant activity	Increased
Red blood cell biomarkers		
Glycophorin A	Integrin‐associated protein. It down‐regulates during RBC senescence	Down‐regulated
CD47	Thrombospondin receptor. It acts as a marker of self	Down‐regulated
PS externalization	Phospholipid, marker of RBC ageing and death, when externalized to the outer leaflet of the plasma membrane	Increased percentage of RBC with externalized PS
Platelet biomarkers		
PS externalization and loss of mitochondrial membrane potential	Pro‐coagulant	Detected
Mitochondrial membrane hyperpolarization without PS externalization	Potentially pro‐coagulant	Detected

NT‐proBNP: N‐terminal pro‐brain natriuretic peptide; MPO: myeloperoxidase; ESR: erythrocyte sedimentation rate; PAP: plasma antioxidant power; ADMA: asymmetric dymethylarginine; sP‐selectin: soluble P‐selectin; sAnnexin V: soluble Annexin V; PS: phosphatidylserine.

## Limitations

Being a rare disease, the evaluation of KD pathogenesis, is extremely complex because it is deeply affected by the small number of patients, the small sample size (biological samples), the peculiarity of the affected patients (infants and children), the collection of limited laboratory data as well as the lack of multicenter collaboration. In addition, KD patients share their features, *e.g*. inflammatory markers, with several other chronic and acute diseases. The consequent absence of a pathognomonic laboratory test allows the diagnosis of KD depending almost exclusively on recognition of clinical criteria, slowing down or often losing the chance of monitoring and identifying suitable KD pathogenetic determinants.

## Future directions

Despite several decades of research, the aetiology of KD remains elusive. Specific basic research and clinical studies should be undertaken to optimize KD clinical diagnosis based on corroborated laboratory data, allowing the compilation of universally suitable guidelines. Laboratory analyses could then allow an earlier and focused diagnosis, and prompting treatments also improving appropriateness of the cures. These aims could be reached by: (*i*) supporting a more stringent clinical surveillance, (*ii*) creating multicenter clinical networks facilitating enrolment of patients with this uncommon disease and efficiently test for new therapies aimed at preventing the disease progression, (*iii*) creating database registries for long‐term follow‐up of KD patients, (*iv*) improving clinical studies aimed at discovering the aetiology of KD and (*v*) deciphering genetic influences on disease susceptibility and outcome.

## Conclusions

This review shows a complex framework of events contributing to the etiopathogenesis of KD. These include some type of bacterial or viral infection, genetic determinants, immune system as well as haematological alterations. Altogether these factors can play a role in the onset and progression of the disease. In fact, all these determinants or some of them, when acting synergistically, could give rise to the disease but, in addition, they can also be considered as useful tools for diagnostic and prognostic purposes. Hence, at least as haematological manifestations of KD are concern, we are far from the understanding of the complexity and the intertwining of the cellular and molecular cross‐talk leading to the cardiovascular risk in these patients. Furthermore, in this scenario, genetic predisposition seems to represent a key background capable of impacting on clinical outcome of KD.

## Conflicts of interest

No conflict of interest.
